# Case Report: A rare case of chest wall thyroid carcinosarcoma

**DOI:** 10.3389/fonc.2025.1684784

**Published:** 2026-01-15

**Authors:** Tianyun Wang, Chengpeng Sun, Yuxi Wang, Chunmei Zhao, Minmin Guo, Xiaofang Wang, Xiang Kui, Yan Wang

**Affiliations:** 1 Department of Pathology, The Second Affiliated Hospital of Kunming Medical University, Kunming, Yunnan, China; 2 Department of Surgery, Yunnan Maternal and Child Health Hospital, Kunming, Yunnan, China

**Keywords:** carcinosarcoma, metastasis, papillary thyroid carcinoma, radioactive iodine therapy, thyroid

## Abstract

Thyroid carcinosarcoma (TCS) is a rare and aggressive malignant tumor, typically reported as a primary thyroid neoplasm. Here, we present an unusual case of TCS occurring in the chest wall. The patient was a 66-year-old woman with a history of papillary thyroid carcinoma (PTC) diagnosed 12 years prior. She received two courses of radioactive iodine (RAI) therapy (200 mCi and 150 mCi) five years ago for metastatic disease in cervical lymph nodes and lungs. Four years after completing RAI treatment, she presented with a progressively enlarging left chest wall mass, which was surgically resected and pathologically confirmed as TCS. This report details the patient’s clinical course and explores the temporal and topographic association between the development of TCS and the prior RAI therapy.

## Introduction

Thyroid carcinosarcoma (TCS) is a rare and aggressive tumor with both epithelial and mesenchymal differentiation (1). Only 20 cases have been reported, making up less than 1% of all thyroid tumors (2). TCS is not included in the fifth edition of the WHO classification for thyroid tumors and was previously considered a variant of undifferentiated thyroid cancer (3). However, given that TCS is composed of recognizable differentiated thyroid carcinoma and sarcomatous elements, its classification remains a subject of debate. There is no standard treatment for TCS, which currently relies on surgical resection and postoperative radiotherapy. Unfortunately, the disease is highly aggressive, often recurring and metastasizing, with most patients passing away within a year of diagnosis (4).

Based on the cases in the literature, TCS is mostly discovered incidentally in thyroid masses, while diagnosis in chest wall masses may be a form of disease that has never been recorded before. This article describes a female patient who received radioactive iodine (RAI) treatment due to the progression of Papillary thyroid carcinoma (PTC), and was diagnosed with TCS in a chest wall mass four years later. This study, while describing the patient’s disease course, focused on discussing the possible relationship between this special case and RAI treatment, and emphasized the importance of close follow-up and examination after the relevant treatment.

## Case report

**Figure 1:**
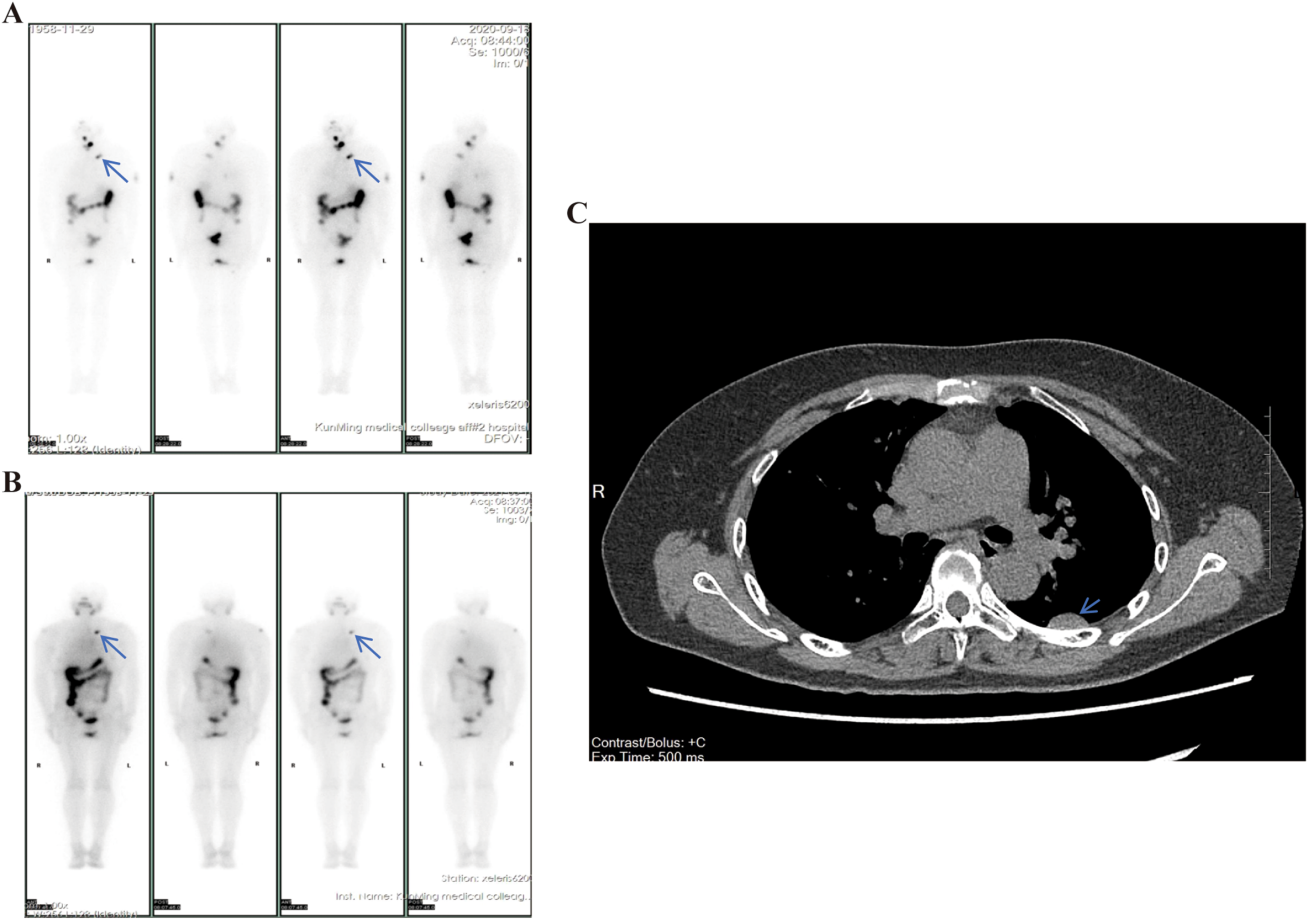
Imaging report. **(A)** Whole-body imaging after the first I-131 treatment showed a mass-like radioactive focus (blue arrow) next to the left sternum, with possible lymph node metastasis. **(B)** Whole-body imaging after the second I-131 treatment showed a mass-like radioactive focus (blue arrow) still visible next to the left sternum. **(C)** Chest CT revealed a mass in the left lower lobe of the lungs (blue arrow) measuring approximately 2.1*1.1 cm.

A 66-year-old woman presented to our hospital with a four-year history of a progressively enlarging mass on the left anterior chest wall. In 2013, the patient had a bilateral thyroidectomy at another hospital for PTC, The initial diagnostic hospital is located far from our institution, and the surgery was performed over ten years ago, so we were unable to obtain detailed records of the first operation or the original pathological samples. After the thyroidectomy, the patient did not undergo RAI treatment. Postoperatively, thyroid hormone tablets were prescribed, but no regular follow-up was conducted. In 2020, the patient noticed a lump on the left side of the neck. Ultrasound examination indicated a recurrence of the malignant thyroid tumor. The patient underwent resection of the lump at our hospital, and histopathological examination of the tissue showed multiple PTC metastases in the cervical lymph nodes. Standardized tissue sampling and sectioning were performed during this diagnostic workup. The pathological examination results suggest a classic morphological PTC lymph node metastasis. Based on the past medical history, the patient should have developed secondary lymph node metastasis on the basis of the previous PTC. During the diagnostic and treatment process, the patient’s chest CT scan revealed multiple masses in both lungs, suggesting metastatic lesions. To treat the metastatic lesions in the lungs and any remaining cancerous lesions, the patient underwent radioactive I-131 therapy. The treatment dose was 200 mCi. Post-treatment whole-body imaging results indicated (1): Significant thyroid uptake remaining in the neck (2); A nodular radioactive accumulation in the left parastern region, suspected to be lymph node metastasis (**Figure 1A**). In 2021, the patient received a second radioactive I-131 therapy with a dose of 150 mCi. The whole-body imaging results after iodine therapy indicated (1): No thyroid uptake remaining in the neck (2); A nodular radioactive accumulation in the left parastern region (**Figure 1B**). During the physical examination, a hard mass of approximately 1 *1 cm was palpable on the left side of the sternum. After the treatment, the patient did not return to our hospital for a follow-up examination as instructed. In 2025, the patient returned to our hospital for treatment due to progressive enlargement of the left chest wall mass over four years, accompanied by left upper limb pain. Specialized physical examination: A mass was palpable on the left chest wall, approximately 8*6 cm in size, firm in consistency, with limited mobility and no tenderness. Laboratory tests: Thyroglobulin > 500 ng/ml. Chest CT (**Figure 1C**): Multiple nodules in both lungs, larger than previously noted, with the largest located in the left lower lobe, measuring 2.1 *1.1 cm, suspected to be metastatic. Given the size and symptomatic nature of the chest wall mass, the patient opted for surgical resection.

**Figure 2:**
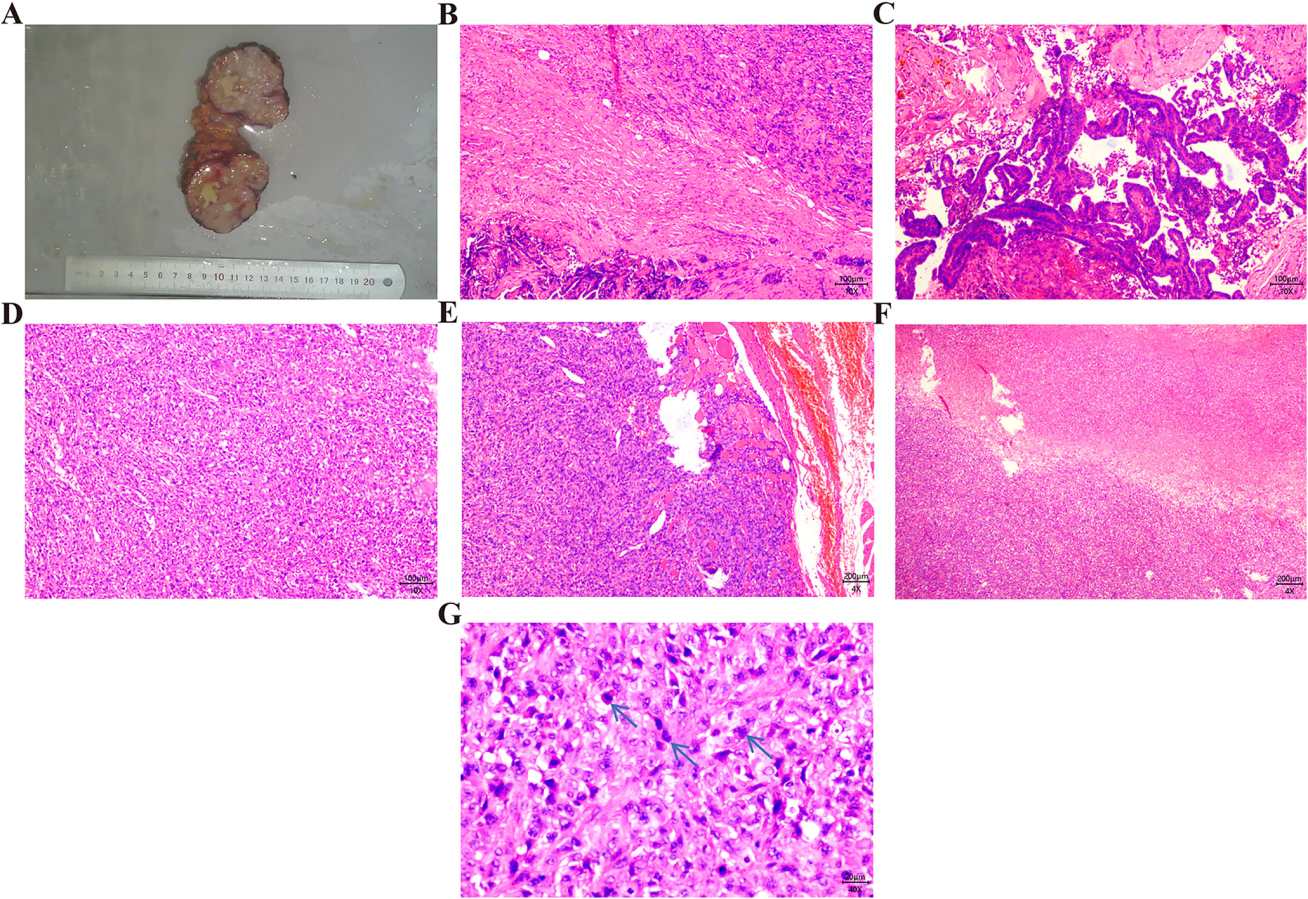
Gross appearance and microscopic characteristics of the tumor. **(A)** This mass appears grayish-white to grayish-yellow and is slightly firm; **(B)** The tumor is made up of two components: sarcomatous tissue in the upper part and epithelial tissue in the lower part, X40; **(C)** The epithelial tissue contains many papillae, with a crowded cell arrangement and minimal atypia, X100; **(D)** The sarcomatous tissue shows numerous spindle-shaped tumor cells arranged in sheets and solid patterns, X100; **(E)** Spindle-shaped tumor cells invade the surrounding striated muscle, X40; **(F)** Extensive tumor necrosis can be seen in the sarcomatous tissue, X40; **(G)** Spindle-shaped tumor cells show peculiar nuclei with prominent nucleoli, and mitotic figures are easily seen (indicated by the blue arrow), X400.

**Figure 3:**
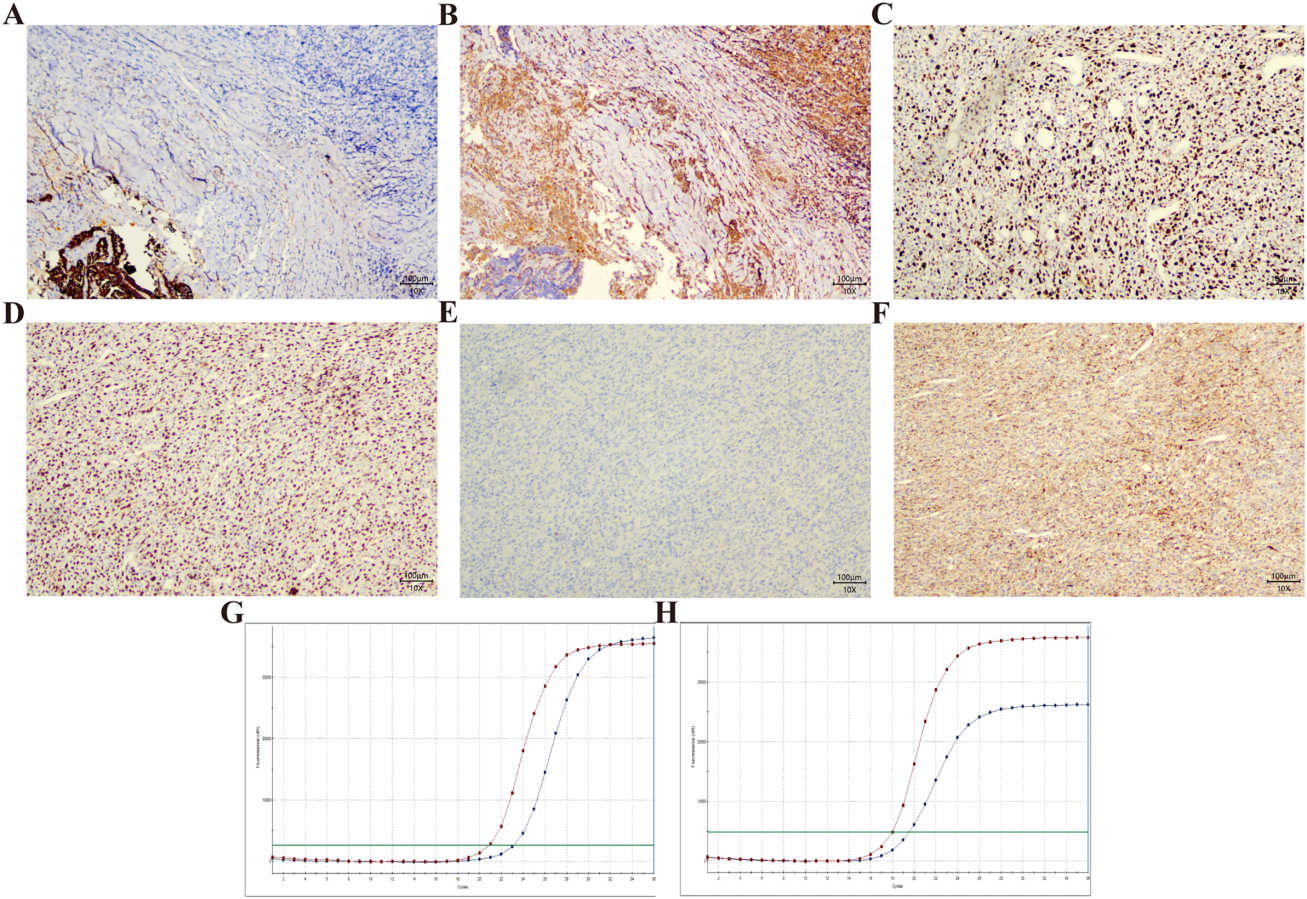
Immunohistochemistry and molecular test results. **(A)** Pan-CK was negative in sarcoma tissue and positive in epithelial tissue; **(B)** Vimentin was positive in sarcoma tissue and negative in epithelial tissue; **(C)** Spindle cells showed strong positive P53 expression; **(D)** Spindle cells show about 90% Ki-67 expression; **(E)** Spindle cells show negative CK19 expression; **(F)** Spindle cells show positive Galectin-3 expression; **(G)** BRAF V600E mutation was found in sarcoma tissue; **(H)** BRAF V600E mutation was found in epithelial tissue.

**Figure 4:**
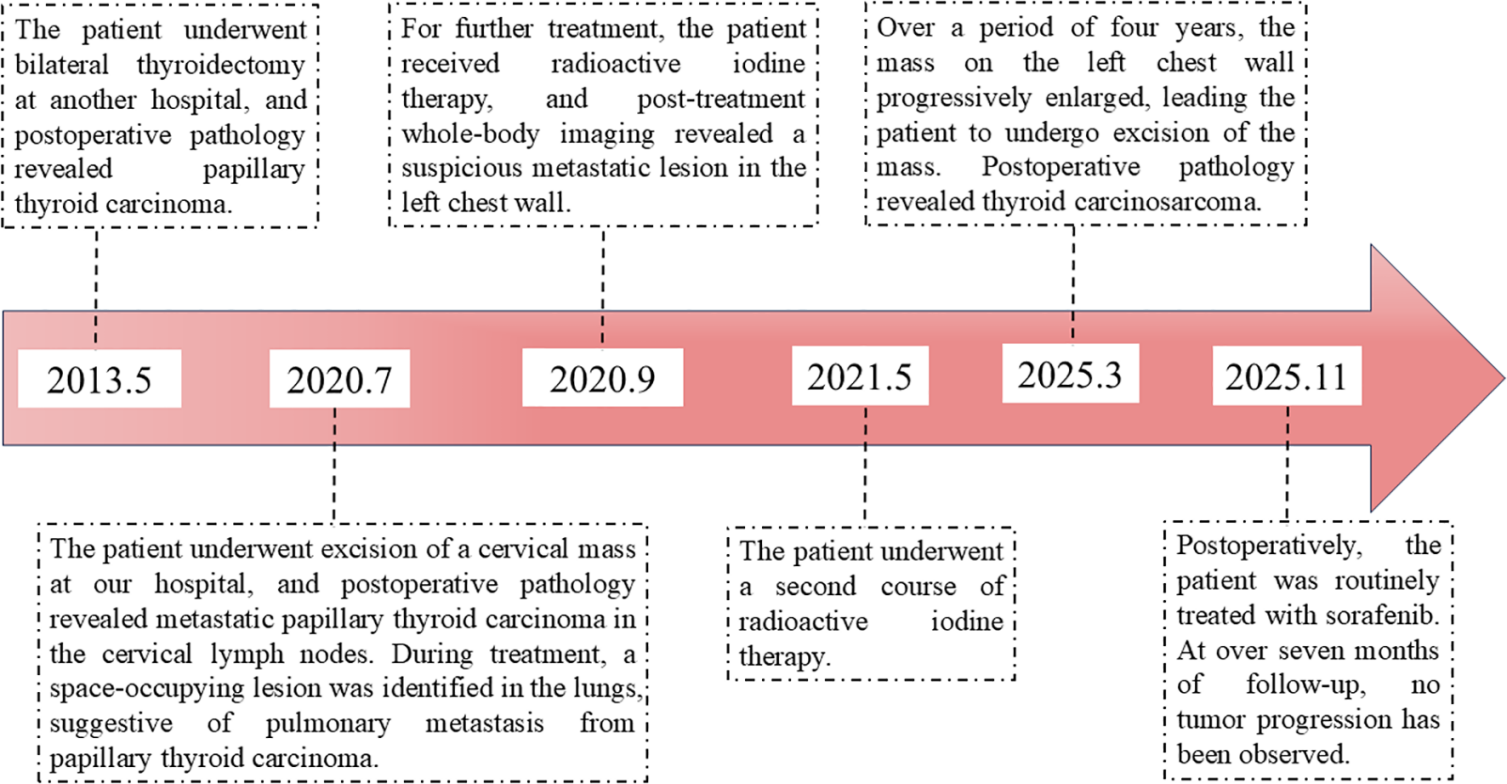
Timeline of disease progression.

Macroscopic findings (**Figure 2A**): The mass has unclear boundaries with surrounding tissues, with a grayish-white to grayish-yellow appearance on the cut surface, a firm texture, and dimensions of 8 *7 *4.5 cm. Pathological findings (**Figures 2B–G**): At low magnification, two different cellular components are seen (1): one resembling PTC, with numerous well-differentiated papillary structures (fibrovascular axes), tightly packed cells, low atypia, and ground-glass nuclei with nuclear grooves (2). The other type consists of spindle cells closely grouped into solid or sheet-like patterns, with clear invasion of striated muscle. The tumor seems to contain fat vacuoles and shows significant necrosis. Tumor cells are short spindle-shaped or polygonal, with a high nucleoplasmic ratio and significant cellular atypia, with irregular nuclei and prominent nucleoli, and pathological mitotic figures frequently seen (>20/10 high-power fields). Immunohistochemistry (**Figures 3A–F**): Pan-CK (sarcoma-, carcinoma+), vimentin (sarcoma+, carcinoma-), Napsin A (sarcoma+, carcinoma-), Ki-67 (sarcoma 90%), P53 (sarcoma+, mutant type), Galectin-3 (sarcoma+, carcinoma+), CK19 (sarcoma-, carcinoma+), TPO (sarcoma-, carcinoma-), PAX-8 (sarcoma-, carcinoma+). Molecular testing results: Molecular tests were carried out on both sarcoma and carcinoma tissues, revealing BRAF V600E mutations in both tissue types (**Figures 3G, H**). Pathological diagnosis: Based on the medical history and morphological features, the diagnosis was TCS. After surgery, the patient underwent continuous treatment with Sorafenib. While on medication, a drug rash developed. At the more than 7 months follow-up, no tumor progression was observed. The timeline in **Figure 4** shows the disease progression of the patient from the first thyroid surgery in 2013 to the most recent follow-up.

## Discussion

Carcinosarcomas are a rare and unique type of malignant tumors that display both epithelial characteristics of carcinoma and mesenchymal features of sarcoma (5). They have a higher risk of recurrence and metastasis compared to carcinoma alone, and the prognosis for patients tends to be worse (6). Carcinosarcomas in the thyroid gland are even rarer, with only a few case reports available. We present a case of a patient with prior PTC who developed TCS in the chest wall several years after treatment with radioactive I-131. This appears to be the first reported case of TCS presenting in the chest wall. Thyroid cancer development has been linked to radiation exposure, with prolonged or high-dose exposure increasing its incidence (7). However, it is unclear whether TCS is also associated with radiation exposure. According to Cahan and Murray’s standardized criteria for radiation-induced sarcoma, the diagnosis of related diseases requires the patient to meet the following criteria (1): radiotherapy followed by the appearance of sarcoma at the site of previous treatment (2); the newly developed sarcoma site shows no relevant lesions prior to radiotherapy (3); the pathological morphology of the newly developed sarcoma must be distinctly different from that of previous tumors (4); A latent period of several years must pass from radiotherapy to the appearance of the sarcoma. In the case we present, the patient underwent bilateral thyroidectomies for PTC 12 years ago and received two courses of radioactive I-131 treatment for cervical lymph node and lung metastasis 5 years ago. The patient developed a progressively enlarging mass on the left chest wall following RAI treatment. The patient then developed a progressively enlarging mass on the left chest wall, a site that had shown radioactive iodine uptake on post-treatment imaging. The HE section of the resected mass revealed both a PTC component and a sarcoma component made up of spindle cells, indicating that the tumor was a carcinosarcoma with bi-directionally differentiated tissues. The diagnosis was further confirmed by immunohistochemical labeling. A similar case has been reported in the literature (8), where a patient with prior PTC developed TCS in the neck several years after RAI treatment. A notable distinction in our case is the location, which may have arisen from a distant metastatic PTC focus.

The temporal and spatial association between RAI therapy and the emergence of the chest wall carcinosarcoma raises the hypothesis that RAI may have contributed to sarcomatoid transformation within a pre-existing metastatic focus. Following the initial RAI treatment, whole-body imaging showed significant radioactive accumulation in the thoracic lesion, indicating that it is a metastatic focus capable of iodine uptake. It is important to note that I-131 is efficiently absorbed by thyroid tissue, including thyroid cancer cells, where it emits beta rays that cause radiation damage to eliminate residual thyroid tissue or destroy cancer cells. I-131’s specific killing ability makes it a standard postoperative therapy for differentiated thyroid cancer (9, 10). This localized, high-dose radiation exposure represents a plausible mechanism that could have induced tumor dedifferentiation or clonal evolution, potentially explaining the lesion’s progressive enlargement in subsequent years. The radiation accumulated from imaging examinations such as CT cannot be overlooked, as it constitutes a significant source of exposure. A single chest CT session typically delivers tens of mGy (11). In contrast, the total dose from the two RAI treatments in this case was in the tens of Gy range (8), over a thousand times higher. Additionally, radiation from imaging is generally systemic and intermittent, without a direct link to the focal progression and worsening of the lesion observed here. In addition, factors such as genetic susceptibility, environmental exposure, and health status are all risk factors for tumor progression. The patient was 50–60 years old at onset, the peak age for sporadic malignant thyroid tumors. No family history of hereditary disease was reported, arguing against a strong genetic link. The patient had no significant environmental exposure history. Environmentally induced progression is typically systemic and gradual, inconsistent with this clinical course. No other major health issues were present besides PTC. These common risk factors are unlikely the primary drivers of progression. Risk factors that affect disease progression are multiple. The distinct feature of this case is the rapid development of the chest wall lesion within years after RAI therapy, showing a strong temporal and spatial connection between the two events. Therefore, we hypothesize that RAI therapy could be a potential high-risk factor for sarcomatous transformation in this specific context.

Subsequent molecular experiments detected BRAF V600E mutations in both sarcoma and carcinoma components. Molecular detection of BRAF V600E is widely used in the diagnosis, prognosis, and targeted therapy of PTC (12), but has been less explored in thyroid sarcomas, with one report linking a BRAF V600E mutation to a thyroid soft tissue sarcoma (13). Compared to traditional external radiation therapy, I-131 delivers a lower radiation dose, with relatively mild side effects, and the incidence of sarcomatoid transformation induced by this treatment is extremely low (14, 15).While I-131 kills thyroid cancer cells through beta rays, it may also damage the cell DNA (16). Continuous DNA damage can cause malignant transformation of cancer cells (17), and some cancer cells may develop sarcomatoid progression. Tumors with BRAF V600E mutation are more aggressive (18), and it is postulated that the mutation can be significantly activated in the radioactive tumor microenvironment and might contribute to sarcomatoid transformation of the tumor under the influence of various factors (19). In this case, BRAF V600E mutations were present in both cancer and sarcoma tissues. This common molecular signature suggests a shared clonal origin and may have played a permissive or driving role in the sarcomatoid transformation.

TCS is an aggressive tumor with a poor prognosis for patients. Compared to common thyroid cancer, it is more likely to recur and metastasize, and patients often die due to local recurrence and lung metastasis, resulting in an average survival of only 5 months after diagnosis (20). Currently, there is no clear consensus or guideline for the treatment of TCS, and most previous reports have focused on total and subtotal thyroidectomy (1). Unlike differentiated thyroid cancer, poorly differentiated tumors like TCS are not responsive to I-131, and radiation is ineffective in killing tumor cells (21). While a TCS patient have benefited from RAI (2), in our case, the development of carcinosarcoma showed a temporal association with RAI treatment. Therefore, the efficacy and safety of I-131 for treating TCS remain uncertain and require further investigation. Radiotherapy is a commonly used adjunct treatment for malignant tumors, and it has been reported that a patient with TCS showed no tumor progression after one year of radiotherapy (4). In another case, the patient received radiotherapy immediately after surgery, but survival was less than 11 months (1), making it uncertain whether TCS patients can benefit from radiotherapy. In this case, the patient was treated with Sorafenib after diagnosis, with only a drug rash as a symptom, and has been followed for over 7 months without tumor progression. Sorafenib significantly improves progression-free survival in patients with RAI-refractory thyroid cancer and is recommended as the first-line systemic therapy for these patients (22, 23). Sorafenib is a multi-targeted tyrosine kinase inhibitor, an oral medication that targets multiple receptors, including VEGFR, PDGFR, Raf, BRAF, and c-KIT, and it can significantly reduce tumor angiogenesis and slow tumor growth and metastasis (24). The patient in this case had a BRAF V600E mutation in both cancer and sarcoma tissues, and Sorafenib may have contributed to tumor progression inhibition by targeting BRAF. The current follow-up period is only 7 months, and we will continue to monitor the patient’s medication and tumor progression.

This study has several inherent limitations that should be acknowledged to better contextualize the reported case and the proposed hypothesis. a) Whole-body imaging performed after RAI therapy revealed pre-existing metastatic lesions in the left chest wall, indicating that tumor foci were present at this location years before the clinical manifestation of TCS. The subsequent progression of this lesion may reflect inadequate follow-up and a lack of targeted local therapy after RAI, rather than proof that RAI induced an entirely new malignancy. b) To substantiate the inference of “RAI-induced sarcomatous transformation,” pathological confirmation would be required that the pre-RAI chest wall tumor was a pure, well-differentiated PTC without any undifferentiated components. This study inherently lacks such pre-RAI histopathological evidence from the lesion. Although post-RAI imaging demonstrated iodine uptake, a feature typically characteristic of differentiated thyroid carcinoma, this functional evidence cannot substitute for histological confirmation. Therefore, we cannot exclude the possibility that a clinically undetectable, microscopic undifferentiated or sarcomatoid component was already present at the time of RAI treatment. Consequently, the proposed association between RAI and sarcomatoid transformation remains speculative, although it is thought-provoking. c) We were unable to obtain the original pathological slides from the patient’s thyroid resection. Although the primary tumor was diagnosed as PTC at the referring hospital, the possibility of undifferentiated components within it cannot be entirely ruled out. The pathological diagnoses at our hospital, a consensus reached by multiple experienced pathologists, confirmed that the lymph node tissue contained only differentiated PTC, while the chest wall mass exhibited clear biphasic differentiation (carcinoma and sarcoma components). d) Although detection of the identical BRAF V600E mutation in both components supports a common clonal origin, this finding is from a single case. The generalizability of this molecular finding and its specific role in RAI-associated sarcomatoid transformation remain unknown and require validation in future studies. e) The clinical follow-up period after sorafenib treatment is relatively short (over 7 months). Although no disease progression was observed, longer-term data are required to fully assess the efficacy and durability of the response in this context. f) As a single case report, this study cannot establish causality. The associations discussed are observational and intended to generate hypotheses for future research. As with any single-case analysis, we cannot entirely exclude the potential influence of unmeasured confounding factors related to the patient’s individual biology or microenvironment on the observed disease course.

## Conclusions

TCS is a highly malignant tumor. Previous reports have mostly seen it in the thyroid gland, but there are no case records of it occurring in the chest wall for the time being. Among the case introduced, the patient had previously suffered from PTC and developed TCS in the chest wall several years after receiving I-131 treatment. This presentation broadens the known spectrum of TCS. In this case, the distinct timing and location of the lesion suggest a possible, though not proven, connection to prior RAI exposure. This observation reinforces the need for careful, long-term monitoring after RAI treatment, particularly when new or unusual findings arise at sites of previous disease. And patients with BRAF V600E mutations may benefit from targeted therapy with Sorafenib. Of course, up to now, the cause of this tumor remains unknown. Further research is needed to explain the mechanism of its occurrence, with the aim of improving treatment methods and the prognosis of patients.

## Data Availability

The original contributions presented in the study are included in the article/supplementary material. Further inquiries can be directed to the corresponding authors.
